# Characteristics of emergency department visits among older adults with hearing difficultly during the COVID19 pandemic in the US

**DOI:** 10.1038/s41598-024-66820-5

**Published:** 2024-07-09

**Authors:** Juebo Yu

**Affiliations:** Department of Otolaryngology, Affiliated Hospital of Yangzhou University, Yangzhou University, No. 45 Taizhou Rd, Guangling District, Yangzhou, 225001 China

**Keywords:** Hearing difficultly, Emergency department, Older adults, Geriatrics, Public health, Quality of life

## Abstract

Hearing difficulty (HD) may be associated with an increased frequency of emergency department (ED) visits among older adults. The COVID-19 pandemic has adversely affected the health of older adults. However, less is known about the characteristics of ED visits by older adults with HD during the pandemic. This study examines the association between self-reported HD severity and ED visits during the pandemic. This population-based cross-sectional study used self-reported data on HD and the characteristics of respondents aged 65 years or older from three cycles of the National Health Interview Survey from 2020 to 2022. Data were analysed from February 23, 2023, to March 22, 2023. The primary outcome was self-reported ED visits in the past 12 months. This study employed generalised linear models to examine the relationship between ED visits (dependent variable) and HD in older adults, and the effect sizes were expressed as rate ratios. Key independent variables included the reasons for ED visit. Covariates such as demographic characteristics and socio-economic status were controlled for to account for potential confounding effects. During the pandemic, older adults with HD commonly visited the ED because of chronic pain (82.8%), frailty (77.9%), trouble falling/staying asleep (73.2%), hypertension (67.4%), and arthritis (60.1%), all of which were 1.5-times more likely in these adults than in those with normal hearing (chronic pain: adjusted rate ratio [ARR], 1.64 [95% CI 1.44–1.93]; frailty: ARR, 1.57 [95% CI 1.16–1.87]; trouble falling/staying asleep: ARR, 1.51 [95% CI 1.21–1.82]; hypertension: ARR, 1.01 [95% CI 0.92–1.23]; arthritis: ARR, 1.39 [95% CI 1.31–1.57]. Older adults with HD were more likely to visit the ED for chronic pain, frailty, trouble falling/staying asleep, hypertension, and arthritis than those with normal hearing during the COVID-19 pandemic. Our findings will be help for healthcare providers to be aware of these potential barriers and to implement strategies to ensure that patients with hearing difficulties can access necessary emergency care effectively.

## Introduction

Individuals with hearing difficulty (HD) face challenges that may lead to higher reliance on emergency department (ED) care^1^. Although previous studies have examined associations between HD and negative healthcare utilisation outcomes, including difficulties in healthcare accessibility and increased hospitalisations, ED visits, and healthcare costs, less is known about ED visits during the coronavirus disease 2019 (COVID-19) pandemic^2–5^.

People with HD may indeed face a higher likelihood of certain health problems compared to those without hearing impairments. Here are some reasons and health issues that are more prevalent or have a higher impact on individuals with HD, such as social isolation, mental health, cognitive decline, physical health issues, communication barriers in healthcare, safety risks, unhealthy lifestyle, economic and employment challenges, self-medication and over-medication, and health conditions. So we wanted to understand what the main needs are for healthcare systems for people with HD after a major public health event, such as the COVID-19 pandemic. Therefore, we wanted to understand what the main needs of people with HD on the healthcare system are after a major public health event, such as the characteristics of emergency department visits during the COVID-19 pandemic. During the COVID-19 pandemic, individuals with HD may have had several reasons for visiting emergency departments. Some of these reasons could be related to the pandemic itself, while others could be due to the exacerbation of pre-existing conditions or the emergence of new health issues. Here are a few potential reasons, supported by our review results provided^1,3,6–9^: 1. The pandemic's social distancing measures could have led to increased feelings of isolation and loneliness, potentially resulting in mental health issues and communication barriers for individuals with HD, prompting visits to emergency departments 0.2.With the shift to telehealth and disruptions in regular healthcare services, individuals with HD might have faced difficulties in accessing necessary care, leading to emergencies that required urgent attention 0.3. The pandemic may have indirectly affected the health of individuals with HD by increasing their risk of other health issues, such as stress-related conditions or complications from chronic diseases. 4. The inability to communicate effectively with healthcare providers due to HD could have resulted in miscommunications and medical errors, necessitating emergency department visits. Individuals with HD, like anyone else, may have visited emergency departments for a range of health concerns unrelated to COVID-19 but potentially exacerbated by the pandemic’s stressors.

In the post-COVID-19 pandemic period, individuals with HD may continue to visit emergency departments for long COVID-19 symptoms, such as fatigue, breathing difficulties, cognitive issues, muscle and joint pain, sleep disturbances, chest pain, heart palpitations, headaches, neuropsychiatric symptoms, neurological symptoms, cardiovascular symptoms, endocrine and metabolic disorders and otolaryngological symptoms^11,12^. Postacute COVID-19 is a syndrome characterised by the persistence of clinical symptoms beyond 4 weeks from the onset of acute symptoms, lasting for at least 12 months^10^. Older people with HD are more susceptible to changes in their internal and external environments or natural changes than those with normal hearing, and need more healthcare and medical interventions. Understanding the characteristics of ED visits by older adults (age ≥ 65 years) with HD during the COVID-19 pandemic can contribute towards reducing potentially inappropriate ED use and guiding clinical decision making and the allocation of healthcare resources for this demographic.

## Methods

### Study sources

The United States (US) Centers for Disease Control and Prevention (CDC) and US Census Bureau conduct the National Health Interview Survey (NHIS), a continuous nationwide health survey of the US noninstitutionalised population, through in-person home interviews. The NHIS is a questionnaire-based study that is conducted prospectively. A stratified, randomised, multistage, probability cluster approach was used to identify households. Health interviews were administered in either English or Spanish at the participants’ homes. The sponsors of each study developed sample weights for each study using data from the US Census Bureau, which employed a multistage area probability sampling design to correct for age, gender, household size, educational level, and race/ethnicity of the most educated household member. All prevalence estimates given in this study reflect the complicated weighting. The NHIS sample designs and methodologies were constant throughout all years.

This study included participants who were at least 65 years old and who answered the Sample Adult questionnaire in 2020 or 2022, which included comprehensive enquiries on demographic attributes and medical history. The NHIS public use files were downloaded from their website. The Research Ethics Review Board of the National Center for Health Statistics approved the NHIS protocol. We examined the primary reason for visit (chief complaint) as reported by the respondent and/or proxy and coded according to the NHIS. The main independent variables were respondent-reported sociodemographic characteristics, frequency of ED visits and self-perceived hearing status. Hearing status was classified as ‘No difficulty’, ‘Some difficulty’, ‘A lot of difficulty’, and ‘Cannot do at all’. The main outcome was the number of respondent-reported ED visits in the past 12 months (including visits to hospital emergency rooms and urgent care centres; ‘how many times have you gone to an urgent care centre or a clinic in a drug store or grocery store due to health concerns?’). The demographic characteristics analysed were age, gender, race and ethnicity, marriage status, educational level, body mass index, family monthly poverty index level, alcohol consumption, smoking status, chronic conditions (asthma, coronary heart disease, diabetes, hypertension, stroke, chronic obstructive pulmonary disease, liver disease, arthritis, and kidney disease), and hearing aid use. Participants in the NHIS provided written informed consent, and study procedures were approved by the National Center for Health Statistics Research Ethics Review Board. Data are deidentified. Therefore, this cross-sectional study was exempted from review by the review board of the Affiliated Hospital of Yangzhou University because it was conducted using publicly available, anonymous data (https://www.cdc.gov/nchs/nhis/data-questionnaires-documentation.htm). Our study was performed in accordance with the Declaration of Helsinki. Data were analysed from May 2, 2023 to October 18, 2023.

### Statistical analysis

Data on categorical variables are presented as frequencies and percentages, whereas data on continuous variables is presented as means and standard deviations. The χ^2^ test and the independent test or Mann–Whitney U test were used to compare categorical and continuous variables, respectively. We used generalised linear models incorporating all covariables to perform multivariable regression for each group of ED visits, the primary outcome being the difference in rate between the prepandemic and postpandemic periods. The effect sizes were expressed as rate ratios and 95% confidence intervals (Cis). Complex survey design methods were used to make annual estimates by appending the 2020–2022 NHIS data. All analyses were performed using R, version 4.0.0 (R Project for Statistical Computing). Statistical significance was defined as a two-sided *P* value < 0.05.

### Consent to participate

This cross-sectional study was deemed exempt from review by affiliated hospital of YangZhou university review board and did not require informed consent because it was conducted using publicly available, deidentified data.

## Results

The NHIS data collected during the specified study period corresponded to 88,701 respondents. We excluded individuals lacking exposure and covariate data to obtain a sample of 27,557 respondents aged 65 years or older, of which 29.7% (8176) had self-reported HD and 25.6% (7052) reported visiting the ED. The summary statistics on the sample’s demographic characteristics are presented in Table 1. Among respondents with self-reported HD, the hearing status of 88.7% (7255) was classified as ‘Some difficulty’, that of 10.8% (882) was classified as ‘A lot of difficulty’, and that of 0.4% (39) was classified as ‘Cannot do at all’. The mean (standard error) age of respondents with self-reported HD was 74.2 (6.4) years. Moreover, 57.3% of the respondents with self-reported HD were men and 42.7% were women; 6.6% were of Hispanic descent, 8.9% were of non-Hispanic and African descent, 3.4% were of non-Hispanic and Asian or Pacific islander descent, 1.2% were of non-Hispanic and native American or Alaska native descent, and 79.4% were of non-Hispanic and European descent. Respondents whose hearing status was classified as ‘A lot of difficulty’ and ‘Cannot do at all’ were more likely to be older, male, and more educated. They were also more likely to report ED visits. Among ED visits by older adults, 2188 visits were estimated to be by individuals with self-reported HD. During the COVID19 pandemic, the common reasons for ED visits by individuals with self-reported HD were chronic pain (82.8%), frailty (77.9%), trouble falling/staying asleep (73.2%), hypertension (67.4%), and arthritis (60.1%), all of which were 1.5-times more likely to occur in these individuals compared with those without normal hearing. The adjusted rate ratios for these conditions were as follows: chronic pain, 1.64 [95% CI 1.44–1.93]; frailty, 1.57 [95% CI 1.16–1.87]; trouble falling/staying asleep, 1.51 [95% CI 1.21–1.82]; hypertension, 1.01 [95% CI 0.92–1.23]; and arthritis, 1.39 [95% CI 1.31–1.57] (Table 2). The common reasons for ED visits by individuals with normal hearing were chronic pain (75.7%), dementia (75.7%), trouble falling/staying asleep (70%), frailty (68%), and hypertension (64%).

**Table 1:** Demographics of respondents by normal hearing and self-reported hearing difficulty severity.

Variable	Respondents^a^			
	Total (%)	Self-reported hearing difficulty (%)		
		Normal hearing (%)	Some of difficulty (%)	A lot of difficulty and cannot do at all (%)
Unweighted sample	27,557 (100)	19,381 (71.6)	7255 (26.3)	921 (2.1)
Age, mean (SD), y	74.15 ± 6.4	73.42 ± 6.2	75.7 ± 6.6	77.59 ± 6.8
Sex				
Male	11,763 (42.7)	7648 (39.5)	3602 (49.6)	513 (55.7)
Female	15,794 (57.3)	11,733 (60.5)	3653 (50.4)	408 (44.3)
Race and ethnicity^b^				
Hispanic (All races)	1832 (6.6)	1401 (7.2)	362 (5.0)	69 (7.5)
Non-Hispanic American Indian or Alaska Native	322 (1.2)	219 (1.1)	88 (1.2)	16 (1.7)
Non-Hispanic White	21,884 (79.4)	14,955 (77.2)	6363 (87.7)	763 (82.8)
Non-Hispanic Black	2463 (8.9)	2002 (10.3)	417 (5.7)	44 (4.8)
Non-Hispanic Asian or pacific Islander	934 (3.4)	712 (3.7)	197 (2.7)	25 (2.7)
Other races	122 (0.4)	92 (0.5)	25 (0.3)	5 (0.5)
Marriage status^c^				
Married	12,245 (46.0)	8627 (46.1)	3245 (46.2)	373 (42.2)
Widowed	5842 (21.9)	3909 (20.9)	1690 (24.1)	243 (27.5)
Divorced or separated	4564 (17.1)	3327 (17.8)	1100 (15.7)	137 (15.5)
Never married or living with other	3965 (14.9	2848 (15.2)	987 (14.1)	130 (14.7)
Smoking status^d^				
Nonsmokers	9790 (36.1)	7116 (45.4)	2399 (40.4)	275 (37.2)
Former smokers	10,207 (37.6)	6805 (43.5)	3010 (50.7)	392 (53.0)
Current smokers	2242 (5.3)	1740 (11.1)	529 (8.9)	73 (9.8)
Alcohol consumption^e^				
Nondrinkers	2251 (12.3)	1625 (12.7)	533 (11.0)	93 (15.7)
Former drinkers	5503 (30.1)	3727 (29.0)	1524 (31.5)	252 (42.4)
Current drinkers	10,520 (57.6)	7490 (58.3)	2781 (57.5)	249 (41.9)
Educational level^f^				
Less than high school	2866 (10.5)	1927 (10.0)	753 (10.4)	186 (20.4)
High school graduate or GED	7370 (26.9)	5070 (26.3)	1992 (27.6)	308 (33.8)
More than high school	17,187 (62.7)	12,292 (63.7)	4479 (62.0)	416 (45.7)
Body Mass Index^g^				
Underweight	472(1.8)	318(1.6)	131(1.8)	23(2.5)
Healthy weight	8569(31.9)	6071(31.3)	2205(30.4)	293(31.8)
Overweight	10,035 (37.3)	7030 (36.3)	2678 (36.9)	327 (35.5)
Obese	7800 (29.0)	5452 (28.1)	2087 (28.8)	261 (28.3)
Income poverty ratio, mean (SD)^h^	3.99 ± 2.82	4.06 ± 2.86	3.92 ± 2.73	3.06 ± 2.31
Hearing aid use				
Yes	4084 (14.8)	1831 (9.4)	1912 (26.4)	341 (37.0)
No	23,473 (85.2)	17,550 (90.6)	5343 (73.6)	580 (63.0)
Emergency department visits				
0 time	17,274 (62.7)	12,327 (63.6)	4430 (61.1)	517 (56.1)
1 time	5157 (18.7)	3622 (18.7)	1348 (18.6)	184 (20.0)
2 times	2496 (9.1)	1714 (8.8)	691 (9.5)	91 (9.9)
3 times	1058 (3.8)	684 (3.5)	333 (4.6)	41 (4.5)
≥ 4 times	1575 (5.7)	1034 (5.3)	453 (6.2)	88 (9.6)
Region				
West	9558 (34.7)	6898 (35.6)	2386 (32.9)	274 (29.8)
Midwest	4368 (15.9)	2924 (15.1)	1277 (17.6)	167 (18.1)
Northeast	3347 (12.1)	2468 (12.7)	791 (10.9)	88 (9.6)
South	10,284 (37.3)	7091 (36.6)	2801 (38.6)	392 (42.6)
Rurality				
Metropolitan	23,630 (85.7)	16,813 (86.7)	6093 (84.0)	724 (78.6)
Nonmetropolitan	3927 (14.3)	2568 (13.3)	1162 (16.0)	197 (21.4)

*GED* General Educational Development.

^a^Data are presented as number (percentage) of respondents unless otherwise indicated.

^b^Indicated as “other” race and ethnicity on the National Health Interview Survey. Hispanic and other race and ethnicity were combined because sample sizes were small.

^c^There were 941 missing unknown values in marriage status.

^d^There were 413 missing unknown values in smoking status.

^e^There were total 18,686 values and 412 missing unknown values in alcohol consumption.

^f^There were 134 missing unknown values in educational level.

^g^BMI, Body Mass Index. For both men and women, underweight is BMI < 18.5; healthy weight is BMI 18.5 to < 25; overweight is BMI >  = 25 to < 30; obese is BMI >  = 30. There were 681 missing unknown values.

^h^Ratio is to the federal poverty level.

**Table 2:** Reasons for visit and diagnoses with ED visits among older adults living with SRHD vs those without SRHD during COVID-19.

Characteristic	No. of visits (% of ED visits)				Rate difference, No. Per 100 (95%CI)^a^	Rate ratio (95%CI)^b^
	Total^1^	SRHD (%)	Total^2^	Non-SRHD(%)		
Reasons for visit among those with SRHD^c^						
COVID-19	1633	155 (9.5)	3368	363 (10.8)	− 1.26 (− 3.05 to 4.78)	0.85 (0.51 to 1.82)
Respiratory tract infection diseases (COPD, Pneumonia, Emphysema, or Chronic bronchitis)	2183	389 (17.8)	4854	614 (12.6)	5.17 (3.31 to 7.03)	1.53 (1.28 to 1.71)
Chronic pain	2039	1688 (82.8)	4530	3427 (75.7)	7.13 (5.07 to 9.20)	1.64 (1.44 to 1.93)
Injury (including, accident, fall, Repetitive Strain Injury, and any injury)	2047	474 (23.2)	4548	892 (19.6)	3.54 (1.38 to 5.70)	1.32 (1.12 to 1.53)
Dementia (including Alzheimer's disease, difficulty remembering/concentrating)	2124	1260 (59.3)	4662	3527 (75.7)	− 16.3 (− 18.8 to − 13.9)	0.52 (0.41 to 0.63)
Frailty	1197	932 (77.9)	2704	1840 (68)	9.81 (6.80 to 12.8)	1.57 (1.16 to 1.87)
Cardiovascular Conditions (including coronary heart disease, angina, stroke, heart attack)	1543	756 (49)	3284	1241 (37.8)	11.2 (8.21 to 14.2)	1.60 (1.40 to 1.79)
Diabetes and Prediabetes	2185	768 (35.1)	4867	1588 (32.6)	2.52 (0.12 to 4.91)	1.12 (0.99 to 1.18)
Trouble falling/staying asleep	1186	869 (73.2)	2640	1845 (70.0)	3.39 (0.32 to 6.45)	1.51 (1.21 to 1.82)
Anxiety	2179	393 (18.0)	4854	698 (14.4)	3.66 (1.80 to 5.55)	1.48 (1.28 to 1.69)
Depression	2180	497 (22.8)	4858	892 (18.4)	4.44 (2.37 to 6.51)	1.64 (1.44 to 1.85)
Weakened immune system (due to prescriptions or health condition)	1692	204 (12.1)	3771	417 (11.1)	0.10 (− 0.85 to 2.85)	0.91 (0.82 to 1.13)
Asthma	2183	346 (15.8)	4863	705 (14.5)	1.35 (− 0.47 to 3.18)	1.29 (0.98 to 1.84)
Difficulty seeing	2182	781 (35.8)	4865	989 (20.3)	15.2 (13.2 to 17.8)	2.26 (2.07 to 2.45)
Hyperlipidemia	2170	940 (43.3)	4838	1810 (37.4)	5.91 (3.41 to 8.39)	1.21 (1.09 to 1.38)
Hypertension	2181	1470 (67.4)	4854	3106 (64.0)	3.41 (1.02 to 5.80)	1.01 (0.92 to 1.23)
Arthritis	2181	1311 (60.1)	4860	2504 (51.5)	8.58 (6.10 to 11.1)	1.39 (1.31 to 1.57)
Cancer	2183	577 (26.4)	4861	1340 (27.6)	− 1.14 (− 3.37 to 1.10)	1.03 (0.94 to 1.12)
Dental disease	1211	233 (19.2)	2727	500 (18.3)	0.91 (− 1.75 to 3.56)	1.12 (0.91 to 1.33)
Delayed counseling/Therapy	2185	140 (6.4)	4867	251 (5.2)	1.25 (0.05 to 2.45)	1.74 (1.43 to 2.26)

*ED* Emergency department, *SRHD* Self-reported Hearing Difficultly, *CI* Confidence interval, *COPD* Chronic Obstructive Pulmonary Disease.

^a^Number of visits among the SRHD group minus number of visits among non-SRHD group per 100 visits.

^b^Adjusted for age(continuous), sex, race and ethnicity, region, education, income, smoking status, drinker status, hearing aids, marital status, BMI, and number of chronic health conditions. Chronic health conditions included hypertension, diabetes, stroke, heart disease, CAD, COPD, anxiety, depression, arthritis, liver condition, chronic pain, dementia, asthma, or cancer.

^c^Reasons for visit represents the chief complaint of the respondent and/or proxy coded according to the 2020–2022 National Health Interview Survey".

^1^Total number of ED visits with SRHD.

^2^Total number of ED visits with non-SRHD.

## Discussion

The COVID-19 pandemic has adversely affected the health of older adults. This epidemiological cross-sectional study is the first to examine the characteristics of ED visits by older adults with HD during the COVID-19 pandemic. Our findings suggest that individuals with self-reported HD commonly visited the ED because of chronic pain, frailty, trouble falling/staying asleep, hypertension, and arthritis. Individuals with normal hearing had similar reasons for visiting the ED, except for the added reason of dementia (including Alzheimer's disease, memory/concentration difficulties).

Our finding that HD was associated with more frequent ED visits is consistent with the results of previous studies^1^. An atypical symptom following recovery from COVID-19 is persistent discomfort, which is comparable to long-term testicular pain. Patients with COVID-19 who have been hospitalised, especially in the intensive care unit, should be concerned about chronic pain^13^. Another review indicates that individuals who experience prolonged pain following COVID-19 of any severity should be given special attention because they are more likely to develop postintensive care syndrome^14^. The prospective risk factors for developing chronic pain following COVID-19 are acute discomfort, prolonged breathing, prolonged immobilisation, neuromuscular inhibition, and neurological injury. The patient’s age and general physical state also influence the likelihood of developing persistent pain after an infection. Individuals who are older or have more underlying medical conditions, especially hypertension, are more likely to experience persistent discomfort after coronavirus therapy. The emotional effects of HD include loneliness, isolation, sedentary lifestyle, depression, and anxiety. Loneliness and a perception of increased social isolation during the COVID‐19 pandemic were associated with increased incidence of all types of pain of different intensities^13,15,16^.

Our results demonstrated that individuals with HD were more likely to visit the ED due to frailty, compared with those with normal hearing. Frailty is marked by a decline in strength, stamina, and physiological function, which makes a person more susceptible to external pressures^17^. It is a heterogeneous condition with a multifactorial aetiology that includes immune, virological, psychological, and endocrine factors^18^. Compromised hearing function may impair the ability to communicate, thus causing loneliness, social isolation, and physical inactivity, which are factors associated with frailty^19^. Decreased physical activity can have various short-term and long-term effects on the body, including weight gain and obesity, joint stiffness, and reduced cardiovascular fitness, muscle mass, strength, bone density, flexibility, and mental well-being.

Trouble falling asleep was another reason why older adults with HD visited the ED. During the initial stages of the COVID-19 pandemic, several research groups and organisations, including the American Academy of Sleep, identified the possibility of the pandemic and the changes it imposed on daily life worsening the quality of sleep because of increased insomnia, increased sleep disturbances, or inconsistent sleep–wake schedules^20^, which is consistent with the results of our study. In addition, individuals with untreated HD often experience difficulties falling asleep or staying asleep throughout the night, probably due to the constant strain of trying to understand sounds and trying to communicate effectively during the day, which leads to increased mental and physical fatigue. Tinnitus can significantly affect sleep quality, making it difficult for individuals to fall asleep or stay asleep. Furthermore, older adults with HD may experience feelings of frustration, anxiety, and depression due to communication difficulties and social isolation, which can, in turn, affect sleep patterns and lead to insomnia or disrupted sleep. For these reasons, older adults with HD are more likely to have sleep problems.

Our study found an association between ED visits and hypertension among older adults with HD during the COVID-19 pandemic. Patients with COVID-19 having hypertension as a comorbidity may require more aggressive treatments, including the use of mechanical ventilation and circulatory support. Viral infection often triggers inflammation and an immune response. Inflammatory factors can increase the risk of hypertension and interfere with its treatment^21^. Therefore, older adults with HD are more vulnerable than the general population.

Interestingly, our study found that during the COVID-19 pandemic, a higher proportion of older people required emergency care for arthritis. However, the specific causes and underlying reasons are still unclear. A recent systematic analysis of case reports and series reported that new‐onset postvaccination polyarthritis, caused by an inflammatory response, was more common in females and older adults^22^. The pathophysiology of COVID-19 and its relationship with arthritis needs to be investigated by determining the viral and antibody titres in the serum and synovial fluid of infected patients, as well as by assessing the incidence and progression of the inflammatory manifestation.

The broader objective of this study is to enable family caregivers, urgent care agencies, and clinical emergency doctors to distinguish between the emergent medical needs of different groups of older adults during the COVID-19 pandemic, so that more accurate help and drug reserves (such as painkillers and antihypertensive drugs) can be provided. This can improve the quality of life and healthy longevity of older adults.

This study has some limitations. First, because the data were self-reported and not obtained through audiometric examination, instances of misreporting and recall bias may be present. Second, we did not determine causation in this study. Finally, potential confounding or selection biases could not be completely controlled for in the study design.

Since 2023, COVID-19 cases have dwindled, but the disease has not been completely eradicated. Our findings can provide insights to clinicians and caregivers about the nature of ED visits among older adults with HD, which can guide clinical decisions and inform the allocation of healthcare resources. Prioritising the health and well-being of older adults during the pandemic is crucial. Measures such as ensuring access to healthcare, providing mental health support, facilitating safe social connections, and prioritising vaccinations for this demographic can help mitigate the adverse effects of COVID-19 on older adults with HD.

## Data Availability

Emergency Department Visits and self-reported Hearing Difficultly data were obtained from the National Health Interview Survey website. (https://www.cdc.gov/nchs/nhis/data-questionnaires-documentation.htm).
